# Comparison of intramedullary nailing and plate fixation in distal tibial fractures with metaphyseal damage: a meta-analysis of randomized controlled trials

**DOI:** 10.1186/s13018-018-1037-1

**Published:** 2019-01-25

**Authors:** Liangcong Hu, Yuan Xiong, Bobin Mi, Adriana C. Panayi, Wu Zhou, Yi Liu, Jing Liu, Hang Xue, Chengcheng Yan, Abudula Abududilibaier, Lang Chen, Guohui Liu

**Affiliations:** 1 Department of Orthopedics, Union Hospital, Tongji Medical College, Huazhong University of Science and Technology, 1277, Jiefang Avenue, Wuhan, 430022 China; 2 Department of Plastic Surgery, Brigham and Women’s Hospital, Harvard Medical School, Boston, 02152 USA

**Keywords:** Distal tibial fracture, Distal metaphyseal fractures, Intramedullary nail, Plate

## Abstract

**Background:**

Distal metadiaphyseal tibial fractures are commonly seen lower limb fractures. Intramedullary nail fixation (IMN) and plate internal fixation (PL) are the two mainstay treatments for tibial fractures, but agreement on the best internal fixation for distal tibial fractures is still controversial. This meta-analysis was designed to compare the success of IMN and PL fixations in the treatment of distal metadiaphyseal tibial fractures, in terms of complications and functional recovery.

**Methods:**

A systematic research of the literature was conducted to identify relevant articles that were published in PubMed, MEDLINE, Embase, the Cochrane Library, SpringerLink, Clinical Trials.gov, and OVID from the database inception to August 2018. All studies comparing the complication rate and functional improvement of I2MN and PL were included. Data on the 12 main outcomes were collected and analyzed using the Review Manager 5.3.

**Results:**

Eleven studies were included in the current meta-analysis. A significant difference in malunion (RR = 1.76, 95%CI 1.21–2.57, *P* = 0.003), superficial infection (RR = 0.29, 95%CI 0.13–0.63, *P* = 0.002), FFI (MD = 0.09, 95%CI 0.01–0.17, *P* = 0.02), and knee pain (RR = 3.85, 95%CI 2.07–7.16, *P* < 0.0001) was noted between the IMN group and PL group. No significant difference was seen in the operation time (MD = − 10.46, 95%CI − 21.69–0.77, *P* = 0.07), radiation time (MD = 7.95, 95%CI − 6.65–22.55, *P* = 0.29), union time (MD = − 0.21, 95%Cl − 0.82–0.40, *P* = 0.49.), nonunion (RR = 2.17,95%CI 0.79–5.99, *P* = 0.15), deep infection (RR = 0.85, 95%CI 0.35–2.06, *P* = 0.72), delay union (RR = 0.92, 95%CI 0.45–1.87, *P* = 0.82), AOFAS (MD 1.26, 95%Cl − 1.19–3.70, *P* = 0.31), and Disability Rating Index in 6 or 12 months (MD = − 3.75, 95%CI − 9.32–1.81, *P* = 0.19, MD = − 17.11, 95%CI − 59.37–25.16, *P* = 0.43, respectively).

**Conclusions:**

Although no significant difference was seen between IMN and PL fixation with regards to the operation time, radiation time, nonunion, deep infection delay union, union time, AOFAS, and Disability Rating Index, significant differences were seen in occurrence of malunion, superficial infection, FFI, and knee pain. Based on this evidence, IMN appears to be a superior choice for functional improvement of the ankle and reduction of postoperative wound superficial infection. PL internal fixation seems to be more advantageous in achieving anatomical reduction and decreasing knee pain.

## Introduction

The optimal type of internal fixation for treatment of a distal radius fracture is still under debate. The tibia is an important weight bearing bone in the lower limb, which articulates proximally with the femur at the knee and distally with the talus at the ankle. Fractures of the distal tibial metaphysis, diaphysis, and adjacent diaphysis are commonly seen in road traffic accidents or sports injuries. These metadiaphyseal fractures are distinct in terms of their management from articular impaction “pilon” type fractures and middle third diaphyseal injuries [1]. The overall incidence of tibial fractures is 51.7 per 100,000 a year, and the incidence of diaphyseal and distal tibia fractures is 15.7 and 9.1 respectively per 100,000 a year [2]. Common definitions of distal tibial fractures include distal extra-articular tibial fractures which are located between 4 and 12 cm from the tibial plafond (AO 42A1 and 43A1). Further subdivisions are made on the basis of the morphology and degree of comminution of the fracture: 43-A1 are non-comminuted extra-articular fractures, 43-A2 are wedge fractures, and 43-A3 are comminuted extra-articular fractures. Simple extension of the fracture into the joint without depression of the joint surface are classified as 43-B1 and are often treated in the same way as 43-A fractures [3–5].

Use of IMN for fracture fixation has been shown that there is limited interference of the device with the soft tissue around the fracture, but the technique of placement is difficult and the learning curve is long. In addition, it has been shown to be linked to complications such as malunion and knee pain after surgery [6–9]. Common surgical procedures for internal fixation of the plate include open reduction and internal fixation and bridge fixation (MIPPO). Open reduction and internal fixation is an anatomical reduction under direct vision, but it is very disturbing to the soft tissue surrounding the fracture. In severe soft tissue injury cases, it is often necessary to extend the preoperative preparation time to optimize soft tissue recovery. Compared with open reduction and internal fixation, MIPPO technology requires fixation with a steel plate, but it also indirect bridge fixation. It is more likely to lead to soft tissue injury than open reduction, but also has a higher rate of fracture malformation and increased local soft tissue pressure possibility [10–13].

The aim of this systematic review and meta-analysis was to compare the efficacy of two fixation methods, plate fixation, and intramedullary nail fixation, in the treatment of distal metadiaphyseal tibial fractures with or without articular involvement.

## Materials and methods

### Search strategy

The databases searched were PubMed, MEDLINE, Embase, the Cochrane Library, SpringerLink, Clinical Trials.gov, and OVID from inception to August 2018. The following search terms were used: distal tibial fracture; intramedullary nail; plate; internal fixation.

### Data selection

To evaluate inclusion eligibility, two investigators independently screened the title and abstracts of all articles. Any disagreements were resolved with discussion between the authors. A third researcher was the adjudicator when there was disagreement between the two investigators. The included studies had to meet the following criteria: (1) must be designed as RCTs; (2) participants must be at least 16 years old; (3) the articles compare intramedullary nail fixation and plate fixation.

### Data extraction

Two authors independently extracted the following data from each eligible study: study design, type of study population, age, number of participants, and interventions. Any discrepancies in data extraction were resolved by a third investigator.

### Quality and risk of bias assessments

The modified Jadad scale was used to assess the methodological quality of each study. A score of ≥ 4 indicated high quality. The Cochrane Handbook for Reviews of Interventions (RevMan Version 5.3) was used to assess the risk of bias. Two independent authors subjectively reviewed all articles and assigned a value of “high,” “low,” or “unclear” based on the following items: selection bias; performance bias; detection bias; attrition bias; reporting bias and other bias. Any disagreements were resolved with discussion to reach a consensus. If a consensus could not be reached a third investigator was consulted.

### Statistical analysis

The RevMan software was used to analyze the numerical data from the included studies. For binary data, the risk ratios (RR), and 95% confidence intervals (CI) were assessed (ɑ = 0.05 for the inspection standards). For continuous data, means and standard deviations (SD) were pooled to a weighted mean difference (WMD) and a 95% confidence internal (CI) in the meta-analysis. Heterogeneity was tested using the *I*^2^ statistic. Studies with an *I*^2^ statistic of 25 to 50% were considered to have low heterogeneity, those with an *I*^2^ statistic of 50 to 75% were considered to have moderate heterogeneity and those with an *I*^2^ statistic > 75% were considered to have high heterogeneity. When the *I*^2^ statistic was > 50%, sensitivity analyses were performed to identify potential sources of heterogeneity. Statistical significance was indicated by a *P* value < 0.05.

## Results

### Description of studies and demographic characteristics

**Fig. 1:**
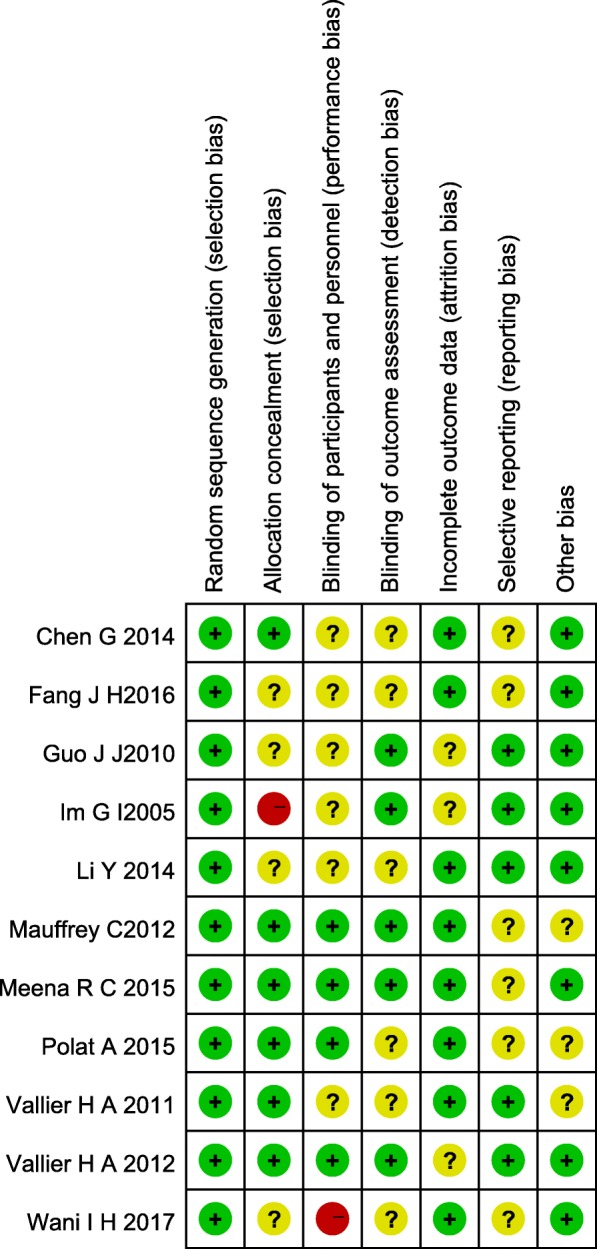
Flow chart showing study identification, inclusion, and exclusion

A total of 889 articles were identified as potentially relevant studies (Fig. 1). A total of 687 full publications were screened based on title and abstracts followed by removal of duplicates (*n* = 202). Twenty full manuscripts were assessed and a further 9 trials were excluded, leaving 11 trials eligible to be included in the meta-analysis.

The demographic characteristics are summarized in Tables 1 and 2. All studies compared postoperative complication rates, postoperative joint function recovery, fracture healing time, delayed fracture healing, wound infection, soft tissue irritation, and postoperative outcomes in the treatment of distal radius fractures in the INM and PL groups. The incidence of knee pain was also extracted from the studies.

**Table 1:** The characteristics of included studies

Study	Year	Country	Patients(n)		Age(Y)		Study Design	Fracture type	Quality Score
			IMN	PL	IMN	PL			
Wani IH [13]	2017	India	30	30	36.4 ± 9.7	38.4 ± 8.7	RCT	OTA42 A1-3	5
Vallier HA [12]	2011	USA	56	48	38.1	38.5	RCT	OTA 42	6
Im GI [15]	2005	Korea	34	30	42(19,65)	40(17,60)	RCT	A1-3,C1	5
Fang JH [18]	2016	China	28	28	35.0 ± 9.2	38.6 ± 7.5	RCT	OTA 42	6
Chen G [19]	2014	China	60	120	53.0 ± 8.1	25.53 ± 8.73	RCT	AO 42A-B	4
Li Y [20]	2014	China	46	46	44(18-78)	43(18-79)	RCT	OTA42	6
Mauffrey C [21]	2012	UK	12	12	50(39-60)	33(24-43)	RCT	EAFDT	6
Guo JJ [16]	2010	China	44	41	44.2(27-70)	44.4(23-69)	RCT	OTA43A1-3	6
Costa ML [22]	2017	UK	161	160	44.3 ± 16.3	45.8 ± 16.3	RCT	EAFDT	6
Polat A [14]	2015	Turkey	10	15	34.0 ± 9.7	36.4 ± 10.7	RCT	OA42/43A1	6
Vallier HA [17]	2012	USA	45	41	41.0	37.8	RCT	OTA42	5

*EAFDT* extra-articular fracture of distal tibia fracture, *OTA* Orthopaedic Trauma Association.

**Table 2:** Characteristics of the eleven trials selected showing general information

Study	TSC	GAT	Gender (F/M)	internal fixation methods		Assessment methods	Follow-up Interval
				IMN	PL		
Wani IH [13]	–	–	18/44	interlocking intramedullary nailing	MIPPO	FFI; union time; weight bearing time; mulunion (rotation; coronal plane; sagittal plane); superficial infection;	3w,6w,9w,12w,3 m,6 m, 9 m,12 m,15 m,18 m; 1y
Vallier HA [12]	–	1,2,3A	19/85	Tibial nail Intramedullary nail	tibial plate no locking plate	Union; malunion; nonunion; infection; secondary operations radiographs	> 12 m,7w
Im G I [15]	C1,2	I	18/46	Intramedullary nail	anatomic plate and screws	Union; malunion; nonunion; angulation, roentgenographic views; wound complications; range of the ankle dorsiflexion; Olerud-Molander ankle score	2y
Fang JH [18]	0 -1	I,II	19/40	interlocked intramedullary; Static locking; primary dynamic locking unreamed tibial nail	distal tibia locking plate	Time: (Operative, Follow-up, Radiation, Bone union, recovery to work) Union; malunion; delayed union; nonunion; Wound complications; pin-tract infection; anterior knee pain; Secondary procedures	>1y,6w
Chen G [19]	–	–	61/95	percutaneous closed reduction interlocking intramedullary nail	Open reduction plate; Percutaneous closed reduction locking compression plate	Union time; operation time; length of incision; radiation time; Radiographic assessment; complication; Union status	1 m,2 m,3 m,6 m.12 m,18 m,24 m
Li Y [20]	–	I,II	13/79	locking intramedullary nail; reamed nail and static locking	minimally invasive plate osteosynthesis	Hospital Stay; OperationTime; Time to radiographic Union; Delayed Union, malunion, Nonunion; Infection (Soft tissue infection, Deep infection, Pin tract infection); Incidence of reoperation; Ankle function	2w,6w,12w,26w,52w
Mauffrey C [21]	–	I	8/16	intramedullary nailing; non-locking screws	locking-plate bridge-plating	DRI; OMAS; EuroQol EQ-5D generalised health outcome questionnaire; union time; Mal-or nonunion	6w,3 m,6 m,12 m
Guo JJ [16]	–	I	35/50	closed intramedullary nailing; Static locking	percutaneous locked compression plate;Static locking	Healing, nonunion; complication; questionnaire concerning removal of the implants for completion by the patients; AOFAS; implant removal questionnaire	6w,3 m,6 m,12 m
Costa M L [22]	–	–	104/321	intramedullary nail	locking plate	later complications; union, nonunion Radiographs; DRI (EQ-5D-3 L, OMAS); Local complications (infection, vascular and neurological injury, venous thromboembolism, malunion); systemic complications; unrelated adverse events	6w,3 m,6 m,12 m
Polat A [14]	–	–	9/16	IMN	MIPO, no blocking screws	Foot function index; time to weight bearing; union time; duration of operation; length of incision; intra-operative blood loss; intra-operative fluoroscopy time; rotational and angular malalignment; rate of infection; secondary interventions; complications	3w,3-6 m
Vallier H A [17]	–	–	13/73	reamed intramedullary nailing	eduction and standard large fragment medial plating	Infections; malunions; nonunions; secondary procedures; Employment status; knee pain; ankle pain; and use of pain Medications;FFI; MFA; general health status measure;	22 m(12 m-71 m)

*IMN* intramedullary nail, *PL* plate, *PTP* proximal tibial plating, *MIPO* Minimally invasive plate osteosynthesis, *DTF* distal tibial fracture, *EAFDT* extra-articular fracture of distal tibia fracture, *OTA* Orthopaedic Trauma Association, *GAT* Gustilo and Anderson Type, *TSC* Tscherne classification, *OMAS* the Olerud and Molander Ankle Score, *AOFAS* The American Orthopaedic Foot and Ankle surgery, *DRI* Disability Rating Index, *EQ-5D-3 L* the EuroQol Health-Related Quality-of-Life 3-Level score, *FFI* Foot Function Index, *MFA* Musculoskeletal Function Assessment, *F/M* female/male, “—” indicates not reported by the study.

### Risk of bias in included studies

**Fig. 2:**
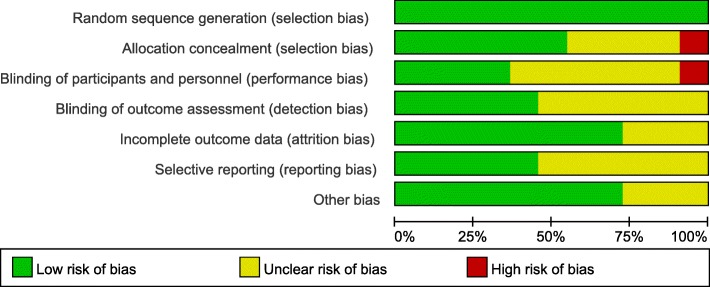
Risk of bias summary

Assessment of risk of bias is presented in Fig. 2. All trials included in this study are randomized trial designs [12, 13]. One trial [14] did not provide detailed information of random sequence generation, and one trial [15] did not describe the method of concealing group allocation. Blinding of participants and personnel (performance bias) was unclear and incomplete outcome data (attrition bias) was high risk in two trials [15, 16]. Three trials [15–17] lost patients to follow-up.

### Operation, radiation, and union time

#### Operation time

**Fig. 3:**
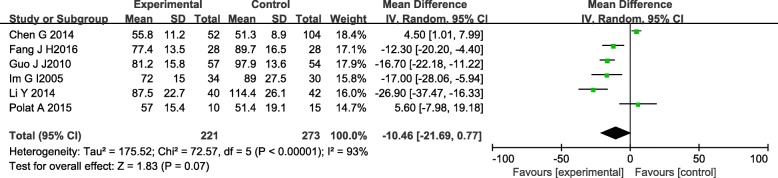
Forest plot of operation time in the IMN group compared with the PL group

Six studies [14–16, 18–20] with a total of 494 patients in both groups provided data on operative time. Heterogeneity in these studies was large, and the random-effects model was used (*I*^2^ = 93%). The meta-analysis showed no significant difference in operative time between the IMN group compared with PL group (MD = − 10.46, 95%CI − 21.69 to 0.77, *P* = 0.07) (Fig. 3).

#### Radiation time

**Fig. 4:**
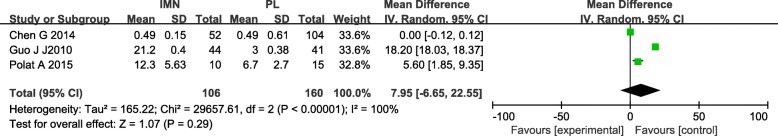
Forest plot of radiation time in the IMN group compared with the PL group

Appropriate data on radiation time were available in 3 articles [14, 16, 19] with a total of 366 patients. Polat et al. [14] recorded the radiation time in milliseconds, whereas the other two study records in minutes, so the comparison could only be done once the time was converted to minutes. There was significant heterogeneity among the studies requiring analysis with a random-effects model (*I*^2^ = 100%). Meta-analysis showed no significant difference in radiation time between the IMN group and the PL group (MD = 7.95, 95%CI − 6.65 to 22.55, *P* = 0.29) (Fig. 4).

#### Union time

**Fig. 5:**
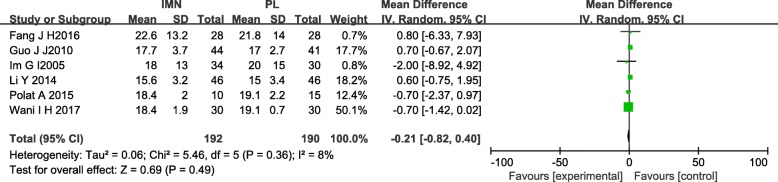
Forest plot of union time in the IMN group compared with the PL group

Six studies [13–16, 18, 20] reported data on union time in the IMN group compared with the PL group. No significant difference in union time was noted between the IMN group and the PL group (MD = − 0.21, 95%Cl − 0.82 to 0.40, *P* = 0.49) (Fig. 5).

### Complication

#### Nonunion

**Fig. 6:**
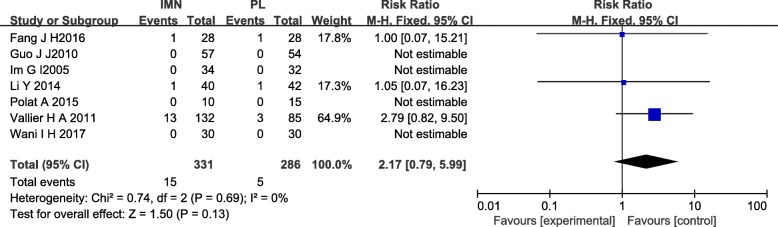
Forest plot of nonunion in the IMN group compared with the PL group

Seven studies [12–16, 18, 20] provided data on nonunion. There was no significant difference in the nonunion rate between the IMN group and the PL group (RR = 2.17, 95%CI 0.79 to 5.99, *P* = 0.15) (Fig. 6).

#### Deep infection

**Fig. 7:**
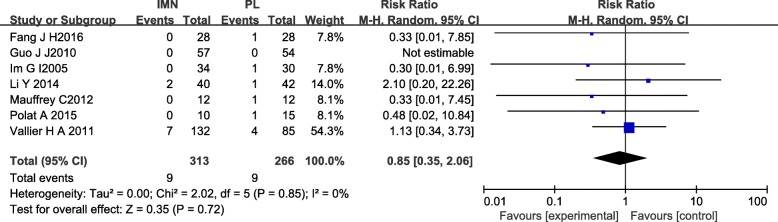
Forest plot of deep infection in the IMN group compared with the PL group

Six studies [12, 14–16, 18, 20, 21] with a total of 579 patients in both groups reported deep infection. There was no heterogeneity among these studies (*I*^2^ = 0%). Data were pooled using the random-effects model and the meta-analysis indicated that there was no significant difference in deep infection occurrence between IMN and PL groups. (RR = 0.85, 95%CI 0.35 to 2.06, *P* = 0.72) (Fig. 7).

#### Malunion

**Fig. 8:**
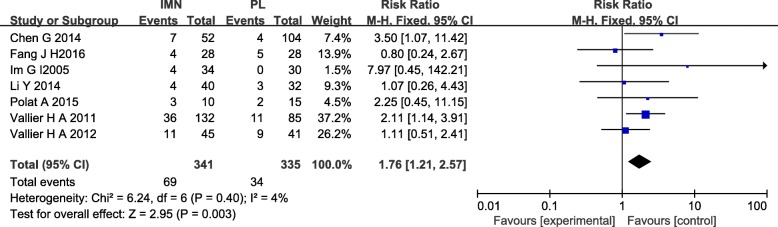
Forest plot of malunion in the IMN group compared with the PL group

Seven studies [12, 14, 15, 17–20] with a total of 676 patients in both groups reported malunion. Mild heterogeneity existed among these studies (*I*^2^ = 4%). Data were pooled by fixed-effects analysis and the meta-analysis indicated that the IMN group had significantly higher malunion versus the PL group. (RR = 1.76, 95%CI 1.21 to 2.57, *P* = 0.003) (Fig. 8).

#### Knee pain

**Fig. 9:**
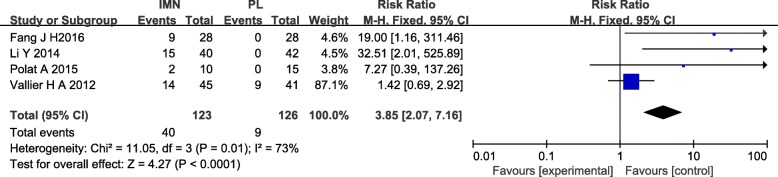
Forest plot of knee pain in the IMN group compared with the PL group

Four studies [14, 17, 18, 20] of 249 patients in both groups reported on knee pain. Heterogeneity was substantial heterogeneity among studies (*I*^2^ = 53%). Data were pooled using a fixed-effects analysis, and the meta-analysis indicated that the IMN group had significantly higher knee pain versus the PL group (RR = 3.85, 95%CI 2.07 to 7.16, *P* < 0.0001) (Fig. 9).

#### Superficial infection

**Fig. 10:**
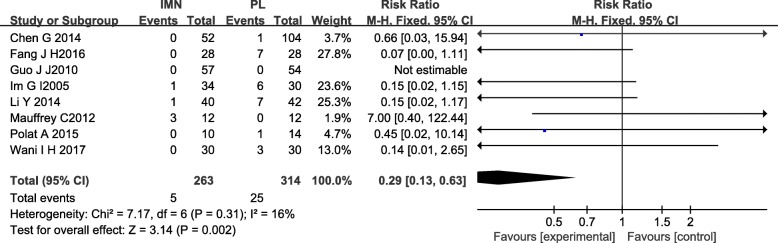
Forest plot of superficial infection in the IMN group compared with the PL group

Eight studies [13–16, 18–21] of 577 patients in both groups reported on superficial infection. There was no heterogeneity among the studies (*I*^2^ = 16%). Data were pooled using a fixed-effects analysis, and the meta-analysis indicated that the IMN group had significantly lower superficial infection rates compared to the PL group (RR = 0.29, 95%CI 0.13 to 0.63, *P* = 0.002) (Fig. 10).

#### Delay union

**Fig. 11:**
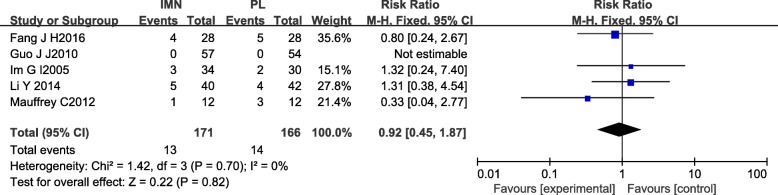
Forest plot of delay union in the IMN group compared with the PL group

Five studies [15, 16, 18, 20, 21] of 337 patients in both groups provided data on delayed union. There was no heterogeneity among studies (*I*^2^ = 0%). Data were pooled using the fixed-effects model, and the meta-analysis indicated no significant difference between the two groups (RR = 0.92,95%CI 0.45 to 1.87, *P* = 0.82) (Fig. 11).

### Objective score

#### American Orthopaedic Foot and Ankle Surgery (AOFAS) score at the 6-month follow-up

**Fig. 12:**
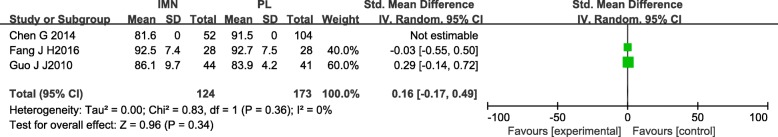
Forest plot of AOFAS score in the IMN group compared with the PL group

Three studies [16, 18, 19] of 297 patients in both groups provided data on the AOFAS at 6 months follow-up. A significant difference in the AOFAS score was noted between the PRP group and the HA group (MD 1.26, 95%Cl − 1.19 − 3.70, *P* = 0.31). However, this result should be interpreted with caution due to presence of low or insignificant statistical heterogeneity (*χ*^*2*^ = 0.88, *I*^2^ = 0%) (Fig. 12).

#### Foot Function Index

**Fig. 13:**
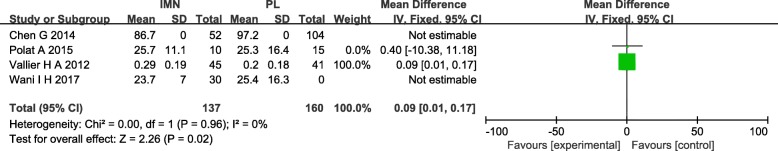
Forest plot of Foot Function Index in the IMN group compared with the PL group

Four studies [13, 14, 17, 19] comprising of 297 patients in both groups provided date on the Foot Function Index. Low heterogeneity among the studies indicated use of the fixed-effects model (*I*^2^ = 0%). The meta-analysis found a significant difference in the Foot Function Index between the IMN group compared to the PL group (MD = 0.09, 95%CI 0.01 to 0.17, *P* = 0.02) (Fig. 13).

#### Disability rating index

**Fig. 14:**
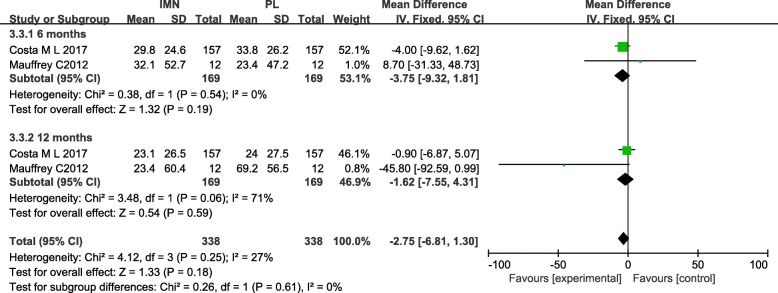
Forest plot of Disability Rating Index in the IMN group compared with the PL group

Two studies [21, 22] focusing on 338 patients in both groups, reported on the Disability Rating Index. Low heterogeneity among the studies at the 6 month time point required adoption of the fixed-effects model (*I*^2^ = 0%). The meta-analysis showed no significant difference in the Disability Rating Index between the IMN group compared with the PL group (MD = − 3.75, 95%CI − 9.32 to 1.81, *P* = 0.19) (Fig. 14). High heterogeneity among the studies at the 12 month time point required adoption of the random-effects model (*I*^2^ = 71%). Meta-analysis showed no significant difference in the Disability Rating Index between the IMN group and the PL group (MD = − 17.11, 95%CI − 59.37 to 25.16, *P* = 0.43). Low heterogeneity among the studies in the subgroup analysis indicated use of the fixed-effects model (*I*^2^ = 27%) and no statistically significant difference was noted (MD = − 2.92,95%CI − 8.71 to 2.87, *P* = 0.32) (Fig. 14).

## Discussion

The earliest PubMed record describing the use of intramedullary nails in the treatment of fractures was a 1946 paper by Otoole reporting the treatment of femoral fractures [23]; use was later reported in the treatment of tibiofibular fractures [24], and subsequently widely applied in limb lengthening. Even in irregular bones such as clavicle fractures, the bone is characterized by highest protection of periosteal blood supply and lowest soft tissue irritation at the fracture end, thus providing a favorable soft tissue environment for fracture healing. IMN can be either reamed or non-reamed, where reaming is more beneficial for reduction. Animal studies have confirmed that there is no significant increase in blood perfusion and osteophyte strength at the fracture end when comparing reamed with unreamed IMN [25]. However, stress occlusion of the fracture end can lead to destruction of local fracture integrity and lead to negative clinical outcomes, occurrences which are expected to decrease with improvement of instrument design techniques and surgery [26]. After the fracture end is reset, the locking nail is locked and pressurized, and the fracture end micro-motion is provided to promote fracture healing, however, if fixed too firmly, the alignment may be poor, the rotation is deformed, or the nail broken [27]. In contrast, open reduction and internal fixation is direct reduction and fixation, so contralateral alignment of the fracture end is superior to IMN fixation. MIPPO is similar to IMN fixation but does not require alignment of the fracture end under direct vision. In addition, it does not require strong fixation and emphasizes soft tissue and blood around the fracture. Limiting the disruption of the blood supply ensures adequate perfusion and maintenance of the biological environment at the fracture ends [28–30]. In addition, treatment of distal radius fractures includes external fixation, external fixation combined with limited open reduction and internal fixation, intramedullary nail and steel plate fixation, but these are not included in the scope of this meta-analysis.

To our knowledge, this is the first meta-analysis looking at the most recent randomized controlled trials comparing the efficacy of IMN and PL in distal tibial fractures and distal metadiaphyseal tibial fractures. This meta-analysis was based on 11 RCTs that included 526 patients treated with IMN and 571 patients treated with PL fixation. The main outcomes investigated were union time, operation time, nonunion, and delay union, superficial infection, deep infection, malunion, and knee pain. In our meta-analysis, when IMN is compared with the PL group, there was no significant difference in AOFAS, delayed union time, deep infection, operation time, and radiation time. However, the IMN group was superior to the PL group in terms of the FFI, knee pain, and malunion. In terms of superficial infection occurrence, the PL group was significantly higher than the IMN group. These results highlight that IMN and PL are effective fixation methods for distal tibial fractures, with no significant differences in fracture healing time and operative time. But the INM group was shown to recover better than the PL group, and the superficial infection rate was lower. However, the malunion and postoperative knee pain in the IMN group were higher than those in the PL group. The operation time, fracture healing time, and intraoperative fluoroscopy time showed no statistically significant difference.

Prior studies [13, 31–33] and several meta-analyses [34–37] investigating the efficacy of IMN have shown that IMN has no superiority over PL when the two methods are compared in well-designed double-blinded trials. The reported beneficial effects of IMN in most trials may have been due to insufficient blinding methods. Our results showed that there was a statistically significant difference in malunion between the IMN and the PL group, with a higher rate of malunion in the IMN group. This is consistent with the results of a prior meta-analysis where no significant difference in operation time and radiation time between INM and PL group was seen (*P* = 0.07, *P* = 0.29, respectively) [34]. This is not consistent with our conclusions. In addition, the limited number of RCTs and patients may also affect these results.

As is the case of many meta-analyses, our study is not devoid of limitations. First, the current meta-analysis focuses only on papers that have been previously published. Inclusion of unpublished research may have increased heterogeneity and changed the current results. Second, the frequency of follow-up varied between studies, with five studies following up for more than 18 months, five studies limited to a year, and one study following up for only 6 months. This variation may affect heterogeneity and hence the results. Furthermore, the type of incision and plate were not consistent across the different studies. There exist several different types of surgical incisions in the use of steel plate fixation, four studies used the MIPPO technique, whereas the rest used open reduction and internal fixation. In terms of type of steel plate, two studies used non-locking steel plates, five used compression-type steel plates, one used an anatomical steel plate, two used a bridge steel plate, and one used ordinary steel plates. Further rigorously designed RCTs, with larger sample sizes, are necessary to better compare the efficacy of IMN and PL fixation.

## Conclusion

The results of this meta-analysis highly support that both the IMN and PL internal fixation methods are effective for the treatment of distal tibial fractures with metaphyseal involvement. However, knee pain has been recorded following use of IMN, malunion is more frequent with IMN fixation, and the risk of superficial wound infection is higher in PL internal fixation than IMN use. In terms of FFI scores, IMN internal fixation appears to be superior to PL. Further studies investigating the use of IMN internal fixation for knee pain after distal tibial fractures are required. Furthermore, the rate of fracture deformity is higher with IMN fixation, while treatment and prevention of superficial wound infections after PL surgery increases the ankle joint function.

## Abbreviations

–: Indicates not reported by the study
AOFAS: The American Orthopaedic Foot and Ankle Surgery
DRI: Disability Rating Index
DTF: Distal tibial fracture
EAFDT: Extra-articular fracture of distal tibia fracture
EQ-5D-3 L: The EuroQol Health-Related Quality-of-Life 3-level score
F/M: Female/male
FFI: Foot Function Index
IMN: Intramedullary nail
KSS: The Knee Society’s Knee Scoring System
MFA: Musculoskeletal Function Assessment
MIPO: Minimally invasive plate osteosynthesis
OMAS: The Olerud and Molander Ankle Score
OTA: Orthopaedic Trauma Association
GAT: Gustilo and Anderson type
PL: Plate
PTP: Proximal tibial plating
TSC: Tscherne classification

## References

[1] Bedi A, Le TT, Karunakar MA (2006). Surgical treatment of nonarticular distal tibia fractures [J]. J Am Acad Orthop Surg.

[2] Wennergren D, Bergdahl C, Ekelund J (2018). Epidemiology and incidence of tibia fractures in the Swedish fracture register [J]. Injury.

[3] Marsh J, Slongo T, Broderick J (2007). Fracture and dislocation classification compendium - 2007: Orthopaedic trauma association classification, database and outcomes committee [J]. J Orthop Trauma.

[4] Newman SD, Mauffrey CP, Krikler S (2011). Distal metadiaphyseal tibial fractures [J]. Injury-international Journal of the Care of the Injured.

[5] Robinson CM, Mclauchlan GJ, Mclean IP (1995). Distal metaphyseal fractures of the tibia with minimal involvement of the ankle. Classification and treatment by locked intramedullary nailing [J]. J Bone Joint Surg Br.

[6] Busse JW, Morton E, Lacchetti C (2008). Current management of tibial shaft fractures: a survey of 450 Canadian orthopedic trauma surgeons [J]. Acta Orthop.

[7] Bode G, Strohm PC, Südkamp NP (2012). Tibial shaft fractures - management and treatment options. A review of the current literature [J]. Acta Chir Orthop Traumatol Cechoslov.

[8] Seyhan M, Unay K, Sener N (2013). Intramedullary nailing versus percutaneous locked plating of distal extra-articular tibial fractures: a retrospective study [J]. Eur J Orthop Surg Traumatol.

[9] Vidović D, Matejčić A, Ivica M (2015). Minimally-invasive plate osteosynthesis in distal tibial fractures: results and complications. Injury-international Journal of the Care of the Injured.

[10] Richard RD, Kubiak E, Horwitz DS (2014). Techniques for the surgical treatment of distal tibia fractures [J]. Orthop Clin N Am.

[11] Gupta RK, Rohilla RK, Sangwan K (2010). Locking plate fixation in distal metaphyseal tibial fractures: series of 79 patients [J]. Int Orthop.

[12] Vallier HA, Beth Ann C, Patterson BM (2011). Randomized, prospective comparison of plate versus intramedullary nail fixation for distal tibia shaft fractures [J]. J Orthop Trauma.

[13] Wani IH, Ul GN, Yaseen M, et al. Operative Management of Distal Tibial Extra-articular Fractures - intramedullary nail versus minimally invasive percutaneous plate Osteosynthesis [J]. Ortop Traumatol Rehabil. 2017:537–41. 10.5604/01.3001.0010.8045 29493524

[14] Polat A, Kose O, Canbora K (2015). Intramedullary nailing versus minimally invasive plate osteosynthesis for distal extra-articular tibial fractures: a prospective randomized clinical trial [J]. J Orthop Sci.

[15] Gun-il I, Suk-Kee T (2005). Distal metaphyseal fractures of tibia: a prospective randomized trial of closed reduction and intramedullary nail versus open reduction and plate and screws fixation [J]. J Trauma.

[16] Guo JJ, Tang N, Yang HL (2010). A prospective, randomised trial comparing closed intramedullary nailing with percutaneous plating in the treatment of distal metaphyseal fractures of the tibia [J]. Ned Tijdschr Traumatol.

[17] Vallier HA, Beth Ann C, Patterson BM (2012). Factors influencing functional outcomes after distal tibia shaft fractures [J]. J Orthop Trauma.

[18] Fang JH, Wu YS, Guo XS (2016). Comparison of 3 minimally invasive methods for distal tibia fractures [J]. Orthopedics.

[19] Chen G, Qian MQ, Zhu GX (2014). Percutaneous closed reduction locking compression plate, percutaneous closed reduction interlocking intramedullary nail and open reduction plate in the treatment of tibial fracture: comparison of biostability [J]. Zhongguo Zuzhi Gongcheng Yanjiu.

[20] Li Y, Jiang X, Guo Q (2014). Treatment of distal tibial shaft fractures by three different surgical methods: a randomized, prospective study [J]. Int Orthop.

[21] Mauffrey C, Mcguinness K, Parsons N (2012). A randomised pilot trial of “locking plate” fixation versus intramedullary nailing for extra-articular fractures of the distal tibia [J]. J Bone Joint Surg Bri.

[22] Costa ML, Achten J, Griffin J (2017). Effect of locking plate fixation vs intramedullary nail fixation on 6-month disability among adults with displaced fracture of the distal tibia: the UK FixDT randomized clinical trial [J]. Jama.

[23] O’toole M (1946). Fracture of shaft of the femur treated with Küntscher intramedullary nail [J]. Nurs Times.

[24] SPRIET (1952). [nailing of fractures of the lower third of the femur and of the tibia by the low approach; introduction of a nail for tibial and condylar malleoli] [J]. Mem Acad Chir.

[25] Schemitsch EH, Kowalski MJ, Swiontkowski MF (1995). Comparison of the effect of reamed and unreamed locked intramedullary nailing on blood flow in the callus and strength of union following fracture of the sheep tibia [J]. J Orthop Res.

[26] Filardi V (2015). The healing stages of an intramedullary implanted tibia: a stress strain comparative analysis of the calcification process [J]. J Orthop.

[27] Lefaivre KA, Pierre G, Holman C (2008). Long-term follow-up of tibial shaft fractures treated with intramedullary nailing [J]. J Orthop Trauma.

[28] Jens F, Geoffroy V, Frank V (2004). Percutaneous plate fixation of fractures of the distal tibia [J]. Acta Orthop Belg.

[29] Borrelli J, Prickett W, Song E (2002). Extraosseous blood supply of the tibia and the effects of different plating techniques: a human cadaveric study [J]. J Orthop Trauma.

[30] Farouk O, Krettek C, Miclau T (1997). Minimally invasive plate osteosynthesis and vascularity: preliminary results of a cadaver injection study [J]. Injury-international Journal of the Care of the Injured.

[31] Chen F, Huang X, Ya Y (2018). Finite element analysis of intramedullary nailing and double locking plate for treating extra-articular proximal tibial fractures [J]. J Orthop Surg Res.

[32] Shen J, Xu J, Tang MJ (2016). Extra-articular distal tibia facture (AO-43A): a retrospective study comparing modified MIPPO with IMN [J]. Injury-international Journal of the Care of the Injured.

[33] Qi Y, Jie N, Li-bin P (2013). Locked plating with minimally invasive percutaneous plate osteosynthesis versus intramedullary nailing of distal extra-articular tibial fracture: a retrospective study. Zhonghua Yi Xue Za Zhi.

[34] Guo C, Ma J, Ma X (2018). Comparing intramedullary nailing and plate fixation for treating distal tibail fractures: A meta-analysis of randomized controlled trials [J]. Int J Surg.

[35] Mao Z, Wang G, Zhang L (2015). Intramedullary nailing versus plating for distal tibia fractures without articular involvement: a meta-analysis [J]. J Orthop Surg Res.

[36] Yu J, Li L, Wang T (2015). Intramedullary nail versus plate treatments for distal tibial fractures: A meta-analysis [J]. Int J Surg.

[37] Kwok CS, Crossman PT, Loizou CL (2014). Plate versus nail for distal tibial fractures: a systematic review and meta-analysis [J]. J Orthop Trauma.

